# Incorporating ecological functions in conservation decision making

**DOI:** 10.1002/ece3.3353

**Published:** 2017-09-07

**Authors:** Emilia Decker, Simon Linke, Virgilio Hermoso, Juergen Geist

**Affiliations:** ^1^ Aquatic Systems Biology Unit Department of Ecology and Ecosystem Management Technical University of Munich Freising Germany; ^2^ Australian Rivers Institute Griffith University Nathan Qld Australia; ^3^ Centre Tecnològic Forestal de Catalunya Lleida Spain

**Keywords:** biodiversity, biological traits, MARXAN, systematic conservation planning, trophic level

## Abstract

Systematic conservation planning has become a standard approach globally, but prioritization of conservation efforts hardly considers species traits in decision making. This can be important for species persistence and thus adequacy of the conservation plan. Here, we developed and validated a novel approach of incorporating trophic information into a systematic conservation planning framework. We demonstrate the benefits of this approach using fish data from Europe's second largest river, the Danube. Our results show that adding trophic information leads to a different spatial configuration of priority areas at no additional cost. This can enhance identification of priority refugia for species in the lower position of the trophic web while simultaneously identifying areas that represent a more diverse species pool. Our methodological approach to incorporating species traits into systematic conservation planning is generally applicable, irrespective of realm, geographical area, and species composition and can potentially lead to more adequate conservation plans.

## 1 INTRODUCTION

Conservation planning can effectively guide decisions about the location, configuration, and management of conservation areas (Margules & Pressey, 2000; Possingham, Wilson, Andelman, & Vynne, 2006). It aims to identify priority areas that comprehensively, adequately, and efficiently protect representative samples of biodiversity. These approaches select areas containing many kinds of biodiversity (=comprehensively) across the full range of variation of each feature (=representative) and enough to ensure persistence of biodiversity (=adequately) for a minimal cost (=efficiently) (Possingham et al., 2006). Conservation often competes with other human interests (Grantham et al., 2013; Lu, Wei‐hua, Zhi‐yun, & Chun‐quan, 2014; Margules, Pressey, & Williams, 2002), forcing trade‐offs to maintain biodiversity. Thus, the science of spatial conservation prioritization aims at identifying locations that are representative of biodiversity at the minimum socio‐economic cost (Moilanen, Wilson, & Possingham, 2009). Once protected areas are established, they provide long‐term security for the biota they cover, maintaining natural processes and viable populations while diminishing direct threats to their biodiversity (Margules & Pressey, 2000; Margules et al., 2002; Wilson et al., 2005).

Systematic conservation planning evolved steadily in the last decades to achieve conservation goals (Cowling & Pressey, 2003; Kirkpatrick, 1983; Rondinini, Wilson, Boitani, Grantham, & Possingham, 2006) and can now be used irrespective of habitat type, geographical area, and species composition (Beger et al., 2010; Watts et al., 2009). To improve adequacy of conservation solutions, that is, “the extent to which reserves fulfil their basic purpose of conserving biodiversity” (Lunney et al., 1997), integrating characteristics of an ecosystem has high priority. Attributes like connectivity (Linke, Pressey, Bailey, & Norris, 2007) and temporal and seasonal variability (Hermoso, Ward, & Kennard, 2012, 2013) are established nowadays and can be applied for prioritizing conservation efforts across all realms. However, other functional attributes such as trophic information are not yet considered in systematic conservation planning.

The trophic level of a species determines its position and therefore its interrelationships (e.g., via competition and predation) with other species in a food web. Taxa at a given trophic level depend on the lower levels as an energy source. Primary producers on the lowest trophic level depend on solar radiation or chemical processes (Lindeman, 1942). The distribution of a species can potentially affect the distribution of other species due to their trophic interactions (e.g., predator–prey relationships) in the food web (Hooker, Whitehead, & Gowans, 2002). Thus, integrating species traits like trophic information in spatial prioritization of conservation efforts should help improve ecologically informed decisions.

Species traits can be subdivided into functional, performance, response, and effect traits (Poff, 1997). They have been used in ecological studies as a biomonitoring tool, in diversity metrics in multivariate adaptive regression splines (Strecker, Olden, Whittier, & Paukert, 2011) as well as factors explaining colonization and invasion success (e.g., Pander, Mueller, Sacher, & Geist, 2016), and could thus also provide an opportunity to make conservation decision making more ecologically robust. However, to the best of our knowledge, species traits related to trophic information have not yet been included in spatial systematic conservation plans.

Food web theory refers to trophic interactions and ultimately determines the fate and flux of every population in an ecosystem (Pimm, Lawton, & Cohen, 1991). Although the benefits for including food web structure to increase adequacy in conservation planning are widely accepted (Dobson, Allesina, Lafferty, & Pascual, 2009), McDonald‐Madden et al. (2016) suggested that the most common measures (e.g., keystone index) of species importance in food webs do not necessarily lead to the best management decisions, as they only take one species into account instead of the entire food web in a more holistic manner. In response, they developed a heuristic approach for prioritizing ecosystem management based on the network wide impact of species protection rather than species loss, showing that considering species interactions can have significant implications for conservation planning (McDonald‐Madden et al., 2016). This is consistent with Stouffer, Sales‐Pardo, Sirer, and Bascompte (2012) suggesting that taking only biodiversity (i.e., overall biodiversity features such as species richness) into account without incorporating species’ ecological function may not be sufficient to preserve an ecosystem's long‐term viability.

Hence, here we test a novel method to incorporate species traits into a spatially explicit conservation plan using trophic level information of fish as an example. We demonstrate the approach using freshwater fish data from a large catchment in Europe, the Danube River. Specifically, we hypothesize that incorporating trophic level information can enhance the adequacy for species in the lower position of the trophic web while simultaneously identifying areas that represent all species, so trophic interactions can be maintained. The method shown in this study is, to our knowledge, the first that addresses trophic information of different species in a systematic conservation plan.

## 2 METHODS

### 2.1 Study area

The Danube River Basin is Europe's second largest river basin, draining catchments across 19 countries and making it the world's most international river basin. Twenty‐seven large (ranging between 4,125 and 157,186 km²) and over 300 smaller tributaries merge into the Danube on its way from the Black Forest to the Black Sea (ICPDR 2015). The delta of the Danube River is one of the world's largest wetlands with 30 different types of ecosystem making the Danube River an important habitat for endemic and endangered species (ICPDR 2015) and a target to achieve favourable conservation status in the context of the Habitats Directive (Council of European Communities 1992).

### 2.2 Planning units

We used HydroBASINS (Lehner & Grill, 2013) as planning unit framework for the conservation planning analyses. HydroBASINS (Lehner & Grill, 2013) is a multiscale set of watershed boundaries and subbasin delineations at a global scale. Fish data were already premapped to Level 8 of HydroBASINS (IUCN, 2012). In total, the Danube catchment was divided into 1,373 subcatchments (= planning units) with 30.73 stream km (ranging between 0.01 and 443.82 km) and contributing areas of 579.18 km² on average (ranging between 0.90 and 7872.40 km²).

### 2.3 Species data

Presence/absence data of fish species in each planning unit were sourced from the BioFresh BioMatrix (IUCN, 2012, last accessed 24 January 2016). All trophic level information, description, and IUCN status of every species were extracted from FishBase (Froese & Pauly, 2015; last accessed 24 January 2016). In total, 128 species occurred in the study area, with an average of 48.0 ± 7.4 species per planning unit. The trophic level of each species was sourced from FishBase with 1.00 being primary producers and detritus feeders and 4.99 indicating high‐level carnivores. The trophic levels of species in our database ranged between 2.00 (=herbivore/detritivore) and 4.5 (= carnivore). For this proof of concept application, species were divided according to their trophic level value into preys (Fishbase trophic level = 2.00–3.99) with 103 species, predators (Fishbase trophic level = 4.00–4.99) with 15 species and a group of 10 species without available trophic level information (Table 1; Appendix S1).

**Table 1:** Division of species into prey and predator group based on trophic level value

Group	Prey		Predator	n.i.
Trophic level value	2.00–2.99	3.00–3.99	4.00–4.99	n.i.
Description	Omnivores, herbivores, detritivores	Mid‐level carnivores	High‐level carnivores	n.i.
Number of species	13	90	15	10

n.i., no information available.

### 2.4 Conservation planning approach

To identify priority areas, the conservation planning software MARXAN (Ball, Possingham, & Watts, 2009) was used. This software aims to solve the reserve design problem known as the “minimum set problem” by finding an optimal reserve network by minimizing cost while maximizing representation of conservation features, and accounting for other aspects such as spatial design (eq. (1)). An important feature in this procedure is the principle of complementarity, which ensures that areas chosen for inclusion in the reserve network complement each other in terms of species composition.

(1) $$\text{Objective function}=\sum_{\mathrm{planning}\;\mathrm{units}}\text{Cost}+\sum_{\mathrm{features}}\text{SPF}\times \text{Feature Penalty}$$

With SPF representing the species penalty factor.

SPF is a weight that applies to the penalties for not achieving the targets for a given species in the final solution. During the optimization, a high SPF (=100.000) for not achieving targets was applied to ensure that all species were adequately represented in the solutions.

Cost shown in equation (1) represents the cost of preserving each planning unit. This is not necessarily the monetary value of a land parcel or stream section, but can also be any relative social (Adams, Mills, Jupiter, & Pressey, 2011), economic (Christensen, Ferdaña, & Steenbeek, 2009) or ecological (Linke et al., 2012) measure of cost, or a combination thereof (Game & Grantham, 2008). Given our interest in exploring the effect of incorporating trophic structure, we used a constant baseline cost across the study area (all the planning units were assigned a cost of 1) similar to Hermoso, Kennard, and Linke (2012). To investigate only the effect of incorporating biological information into the conservation plan, we did not include a connectivity penalty, which is usually used to foster the selection of connected planning units (Hermoso, Linke, Prenda, & Possingham, 2011). We ran the algorithm 100 times with 2 000 000 iterations each and retained the best solution for further analyses.

### 2.5 Incorporating trophic level information in MARXAN

To evaluate the effect of using trophic information in conservation planning, we compared different scenarios in MARXAN that we refer to as “with” and “without trophic information”. In the baseline scenario without including trophic information, the cost of all planning units was homogeneous (cost = 1). For the scenarios with trophic information, cost was discounted relative to the proportion of predator species present in the local assemblage. In this way, a planning unit with a high proportion of predators in the assemblage was assigned a higher cost compared to a planning unit with lower proportion of predator species. Thus, a planning unit with a higher proportion of predators was less likely to be chosen by MARXAN if an alternative was available.

We tested the effect of different trophic weightings in the prioritization process by increasing the importance of the trophic information. Planning units with the highest proportion of predator species were assigned a cost value of 1, while planning units with the lowest proportion of predator species in the assemblage were assigned the lowest cost, which changed accordingly to the scaling range applied in each case. Seven different scaling ranges (0.01–1; 0.5–1; 0.1–1; 0.15–1; 0.2–1; 0.25–1; 0.3–1) (subsequently referred to as “trophic weighting”) were used. An example for this is given in Table 2. Planning unit 4 (=35% predator proportion) was assigned a value of one. Planning unit 3 (=6% predator proportion) was given the lowest value depending on the trophic weighting either 0.3, 0.2, or 0.1.

**Table 2:** Example for different trophic weightings depending on predator proportion

Planning unit	Proportion of predator species (%)	Trophic weighting 0.3–1	Trophic weighting 0.2–1	Trophic weighting 0.1–1
1	20	0.64	0.59	0.53
2	5	0.52	0.45	0.38
3	6	0.30	0.20	0.10
4	35	1.00	1.00	1.00
5	17	0.57	0.50	0.44
6	8	0.35	0.26	0.16

To assess the influence of target size, four different representation targets (3, 5, 7, or 10) in scenarios with and without trophic information were used. This means each species should be represented in 3, 5, 7, and 10 planning units, respectively, within the solution whenever possible. A species must be represented in different planning units, but the same planning units can be accounted for different species at once. Different targets were used to determine interactions between targets and trophic weightings, as large targets lead to less flexibility in the choice of planning units in MARXAN (the larger the targets, the higher the number of planning units needed and then the more similar solutions would be expected to be). The whole series of trophic weightings was used across all targets to ensure a complete analysis of the effect of incorporating trophic level information in conservation planning.

To analyze the overall effect of incorporating trophic level information on the area selected and the average proportion of predator species in the results from MARXAN, the spatial distribution of planning units in the best solution obtained for the scenarios with and without trophic information was compared. We evaluated the spatial overlap between both scenarios by calculating Cohen's kappa coefficient. Further, we compared average proportion of predator species in planning units unique to both scenarios. In this way, we examined the specific effect of incorporating trophic level information without the results being dilute by the proportion of predator species of planning units common in both runs as some planning units were always included in both scenarios to achieve the target of rare species. To examine whether proportion of predators in all scenarios was different from random, we compared the average proportion of predator species of each target and 1000 random samples of the same number of planning units for each solution independently. Statistical analysis was conducted using R (R Core Team 2017). Planning units, river system, and MARXAN solutions were mapped in ArcMap 10.3.1 (ESRI 2016).

## 3 RESULTS

### 3.1 Effect of including trophic level information on area selected

Representation targets were achieved for all 128 species as long as the species occurred in enough planning units. Thirty‐four species occurred in less than 10 planning units making the achievement of that target of 10 unfeasible (Appendix S2), although their whole distribution range was selected.

**Figure 1:**
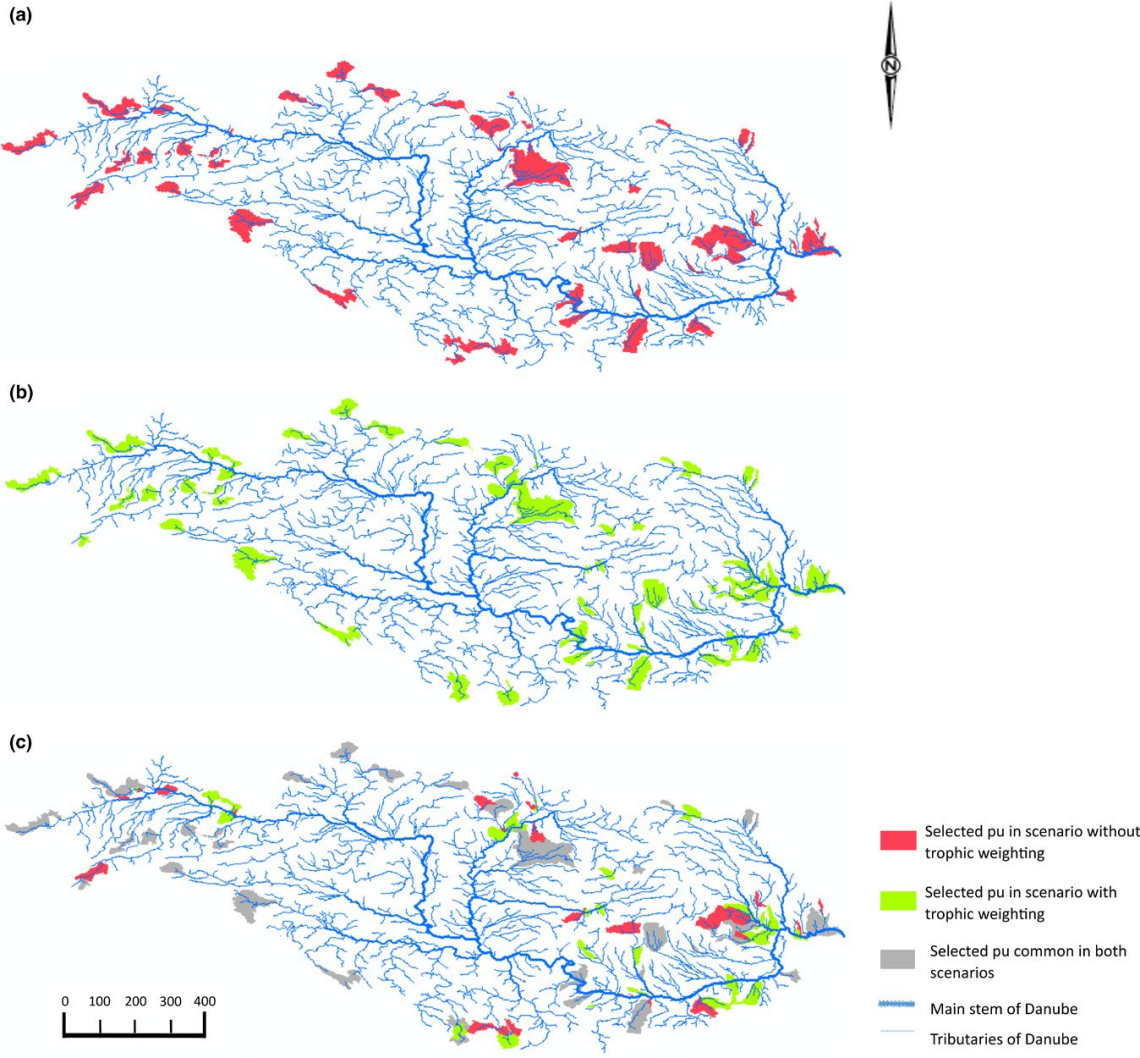
Best solution of planning units with a target of 10 without and with trophic weighting (=scaling 0.01–1). (a) Red‐coloured areas show planning units (pu) selected in scenarios without trophic weighting; (b) green‐coloured areas show planning units selected in scenarios with trophic weighting; (c) difference in selected planning units with (green area) and without (red area) trophic weighting, grey‐coloured areas show planning units selected in both scenarios. Broad blue line indicates main stem; thin blue lines indicate tributaries of the Danube River

Comparing the best solutions across scenarios with and without trophic information (Figure 1a, b, respectively) showed differences in location of selected planning units (Figure 1c), while total number of planning units generally did not change (Table 3). For example, for a target of 3, the exact same number of planning units was chosen in both runs irrespective of the assigned trophic weighting. For the targets 5 and 7—depending on the weighting—zero to one, and for a target of 10, zero to three additional planning units were chosen. Approximately 60% of the planning units selected in both scenarios were identical for all targets used (grey area in Figure 1c, Table 3). The remaining 40% differed and represented the change of planning units selected when trophic information was incorporated (Figure 1c). This was confirmed by Cohen's kappa coefficient indicated 59.8% agreement between all runs with and without trophic information, irrespective of the trophic weighting (*p* < .001) (Appendix S3).

**Table 3:** Number of planning units selected and percentage of common area for each target and trophic weighting

Trophic weighting	Target 3		Target 5		Target 7		Target 10	
	Total area (pu)	Common pu (%)	Total area (pu)	Common pu (%)	Total area (pu)	Common pu (%)	Total area (pu)	Common pu (%)
1	38	100.00	59	100.00	59	100.00	105	100.00
3	38	55.26	60	59.62	59	66.10	106	65.71
4	38	57.89	59	62.71	60	61.02	106	66.67
5	38	57.89	60	59.32	60	57.63	106	65.71
6	38	60.53	60	64.41	60	57.63	106	61.90
10	38	63.16	60	59.32	60	69.49	108	64.76
20	38	57.89	60	61.02	60	57.63	107	60.00
100	38	63.16	60	57.63	60	57.63	107	67.62

Grey highlighted column indicates proportion of common area selected in both runs with and without trophic information. Trophic weighting is ratio highest to lowest with 1 being no weighting (run without trophic information), 3 being a weighting of 0.3–1, 4 being 0.25–1, 5 being 0.2–1, 6 being 0.15–1, 10 being 0.1–1, 20 being 0.05–1, and 100 being 0.01–1.

### 3.2 Effect of including trophic level information on average proportion of predator species

**Figure 2:**
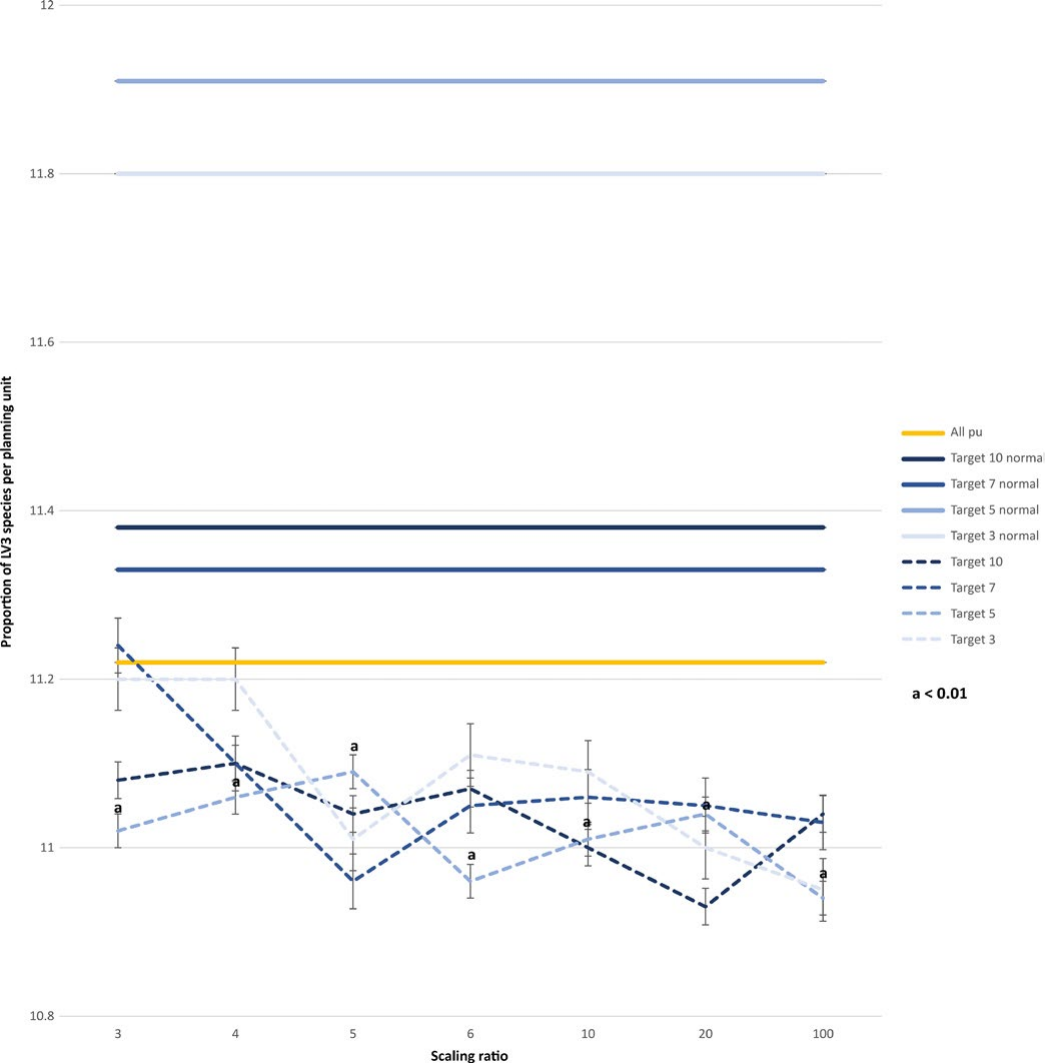
Effect of incorporating trophic level information and different weightings on average proportion of predator species per planning unit in chosen area. Average proportion of predator species for all planning units is indicated by orange line; average proportion of predator species in chosen areas is shown for each target in runs without trophic information and for each target and weighting in runs with trophic information. Proportion of predator species for each target of runs without trophic information are drawn with thicker, solid lines and over all weightings, although no weighting was performed, to illustrate the difference between runs with trophic information, which are drawn with thinner, dashed lines. Trophic weighting is ratio highest to lowest with 3 being a weighting of 0.3–1, 4 being 0.25–1, 5 being 0.2–1, 6 being 0.15–1, 10 being 0.1–1, 20 being 0.05–1, and 100 being 0.01–1; a indicates significant difference (*p* < .001) between scenarios with the same target

**Figure 3:**
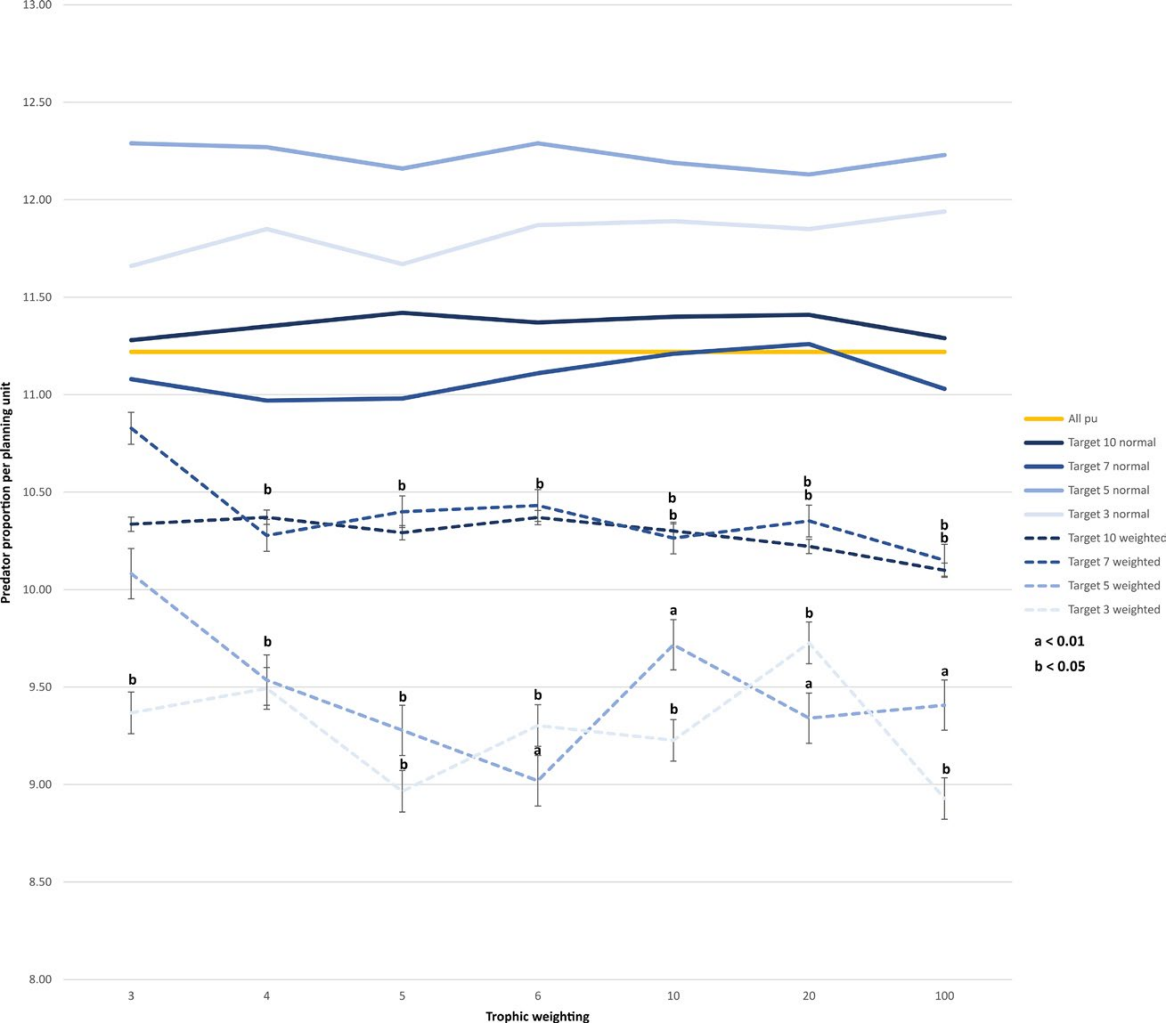
Effect of incorporating trophic level information and different weightings on average proportion of predator species per planning unit in unique chosen area. Average proportion of predator species for all planning units is indicated by orange line; average proportion of predator species in chosen area unique to either runs without or with trophic information is shown for each target and each weighting. Proportion of predator species for runs without trophic information are drawn with thicker solid lines. Proportion of predator species for runs with trophic information are drawn with thinner dashed lines. Trophic weighting is ratio highest to lowest with 3 being a scaling of 0.3–1, 4 being 0.25–1, 5 being 0.2–1, 6 being 0.15–1, 10 being 0.1–1, 20 being 0.05–1, and 100 being 0.01–1; a and b indicate significant difference (*p* < .001 and *p* < .05, respectively) between scenarios with the same target

The average proportion of predator species in the assemblage across the whole catchment was 11.2%. In scenarios without trophic information, the average proportion of predator species in the planning unit selected was 11.6% regardless of the target used. In scenarios with trophic information, the proportion of predators in planning units varied between 10.9% with a low target and high weighting, and 11.2% with a high target and low weighting (Figure 2). Taking only planning units that were unique to each run into account, significant differences between both scenarios became evident. Except for the lowest weighting (0.3–1), runs with trophic information showed a significantly lower proportion of predator species for each target and weighting than runs without trophic information (*p* < .05). The average proportion of predator species in planning units specific to runs without trophic information ranged between 11.0% and 12.3% with targets of 7 and 5, respectively. In the scenario with trophic information, the proportion of predator species varied between 8.9% with a low target and high weighting and 10.8% with a high target and low weighting (Figure 3). Randomization of 1000 samples showed a higher proportion of predators in random samples of the same size than in runs with trophic information (11.28 and 11.26 for targets 3, 5, 7, and 10, respectively), demonstrating MARXAN's choice of planning units being different from random *p* < .001 (Appendix S4).

## 4 DISCUSSION

We demonstrated a novel approach for incorporating biological trait information into cost‐effective conservation planning. This approach can be used irrespective of habitat type, geographical area, and species composition. To our knowledge, this was the first time that trophic information was incorporated in a spatially explicit conservation plan. We incorporated a key ecological dependency between prey and predators, while simultaneously identifying refugia for prey species from predator species. Comparing best solutions of scenarios with and without trophic information revealed approximately 60% concurrence of planning units (Figure 1), leaving 40% of the area chosen by MARXAN adaptable to incorporate biological information. The overlap in both scenarios can be explained by the irreplaceability of certain planning units. Some planning units were always selected as they either contained rare species or had a high diversity, making them irreplaceable for species representation and conservation target achievement. The remaining 40% revealed a significantly lower proportion of predator species in planning units when using trophic information (Figure 3). This demonstrates that our approach provides refugia for prey in places where predator pressure is lower, assuming that the abundance of predators is related to the number of species. Although our conservation features in selected planning units continued to meet the same target in both scenarios, we could incorporate trophic information into MARXAN without increasing the number of planning units by more than one on average (Table 3). Encouragingly, this demonstrates that including ecological function in conservation planning does not necessarily result in an increased area needed for its implementation, but in a more ecologically sound network design.

The effect of including trophic information differed across targets (Figures 2 and 3). With a lower target, MARXAN has more choices between planning units with lower proportion of predator species. If the target is set too high, the effect of the trophic weighting diminishes, as MARXAN is restricted to choose planning units with a higher proportion of predator species to achieve this target. The effect of the trophic weighting depends on its strength. A higher trophic weighting leads to a greater difference between “cheap” and “expensive” planning units, making planning units with lower proportion of predator species more favourable for selection. Thus, depending on the overall conservation goal, trophic weighting and therefore predator pressure in chosen areas can be adjusted.

Using a simplified food web with two groups of species (Table 1), we showed that this type of ecological information can be incorporated into systematic conservation planning, which can make decisions more ecologically robust. Food webs are not steady and experience a constant shift in community dynamic (Polis, Anderson, & Holt, 1997). Therefore, another important part in conservation planning is to monitor the system after conservation actions took place (Margules & Pressey, 2000). We do not offer a detailed conservation plan for an ecosystem, but provide a possible solution that can reduce the risk for prey extinction (Drossel, Higgs, & McKane, 2001). It is likely that including more detailed high‐resolution data on food web complexity and interaction, as well as inclusion of other biological traits, will even result in a better and more robust conservation planning outcome.

Including biological trait information in systematic conservation planning can also be useful to mitigate invasive species which can cause significant impact on native communities and hamper conservation efforts for native species. Within the Danube system, for example, species of non‐native gobies have already strongly altered food web structures resulting in novel community structures (Brandner, Auerswald, Cerwenka, Schliewen, & Geist, 2013; Brandner, Cerwenka, Schliewen, & Geist, 2013) and biological trait information has been linked with colonization and invasion success (Pander et al., 2016). Attempts to eradicate alien species are often unsuccessful or can cause other problems (Bergstrom et al., 2009; Zavaleta, Hobbs, & Mooney, 2001). Thus, our method can be useful to incorporate information about invasive alien species to determine how they may change strategic conservation planning. Using the proportion of invasive species to native ones instead of trophic level information on the proportion of predators to prey in native species only, this method could be applied to predict effects and support conservation management decisions in light of novel community structures that comprise a mix of formerly spatially separated species.

This study should be considered a demonstration exercise on how to incorporate species traits and their effect, rather than producing a detailed conservation plan for the Danube basin. A more realistic conservation plan beyond a demonstration exercise would require the inclusion of other biodiversity information (e.g., number of individuals per species, other freshwater‐dependent species, and ecological processes), information about biophysical processes (e.g., connectivity, disturbance, temporal, and seasonal variability), and a better estimation of conservation or management cost.

## 5 CONCLUSION

We have successfully demonstrated integration of biological traits into a conservation plan using the example of trophic information of fish. With our approach, it is possible to keep costs and species representation constant, while taking their functional traits into account. This study is a first step to accomplish the incorporation of species traits information into systematic conservation planning, paving the way for more realistic and ecological meaningful solutions to conservation issues.

## CONFLICT OF INTEREST

None declared.

## AUTHORSHIP

ED, SL, VH, and JG conceived the project; ED performed the analyses as part of her master thesis under supervision by JG and SL; SL and ED preformed the statistical analyses; ED wrote the first draft of the manuscript; SL, VH, and JG discussed analyses, inferences, and all authors contributed substantially to revisions.
