# Supplementary material for: Incorporating ecological functions in conservation decision making
Source: Ecol Evol. 2017 Sep 7;7(20):8273–81. doi: 10.1002/ece3.3353 (PMC5648659; doi:10.1002/ece3.3353)
Supplement: Supplementary file 1 [file ECE3-7-8273-s001.docx]

**Appendix S1: Species trophic level according to FishBase and assigned group.**

n.i. = no information available

| **Species name** | **trophic level from FishBase** | **group** |  |  |
| --- | --- | --- | --- | --- |
| *Abramis brama* | 3.1 ±0.1 se | prey |  |  |
| *Acipenser gueldenstaedtii* | 3.3 ±0.1 se | prey |  |  |
| *Acipenser nudiventris* | 3.3 ±0.45 se | prey |  |  |
| *Acipenser ruthenus* | 3.6 ±0.43 | prey |  |  |
| *Acipenser stellatus* | 3.5 ±0.2 se | prey |  |  |
| *Acipenser sturio* | 3.5 ±0.51 se | prey |  |  |
| *Alburnoides bipunctatus* | 2.9 ±0.35 | prey |  |  |
| *Alburnus alburnus* | 2.7 ±0.29 se | prey |  |  |
| *Alburnus mento* | 3.0 ±0.2 | prey |  |  |
| *Alburnus sarmaticus* | 3.0 ±0.2 se | prey |  |  |
| *Alosa fallax* | 4.0 ±0.4 | predator |  |  |
| *Alosa immaculata* | 4.0 ±0.6 | predator |  |  |
| *Alosa maeotica* | 4.4 ±0.8 se | predator |  |  |
| *Alosa tanaica* | 3.5 ±0.6 se | prey |  |  |
| *Anguilla anguilla* | 3.6 ±0.3 | prey |  |  |
| *Aspius aspius / Leuciscus aspius* | 4.5 ±0.80 se | predator |  |  |
| *Atherina boyeri* | n.i. | n.i. |  |  |
| *Aulopyge huegelii* | n.i. | n.i. |  |  |
| *Babka gymnotrachelus* | 3.3 ±0.3 se | prey |  |  |
| *Ballerus ballerus* | 3.2 ±0.36 | prey |  |  |
| *Ballerus sapa* | 2.8 ±0.3 | prey |  |  |
| *Barbatula barbatula* | 3.3 ±0.0 | prey |  |  |
| *Barbus balcanicus* | 3.0 ±0.3 | prey |  |  |
| *Barbus barbus* | 3.1 ±0.39 | prey |  |  |
| *Barbus carpathicus* | 2.9 ±0.3 | prey |  |  |
| *Barbus petenyi* | 2.9 ±0.3 | prey |  |  |
| *Benthophiloides brauneri* | 3.4 ±0.49 | prey |  |  |
| *Benthophilus nudus* | 3.4 ±0.5 | prey |  |  |
| *Blicca bjoerkna* | 3.2 ±0.0 | prey |  |  |
| *Carassius carassius* | 3.1 ±0.24 | prey |  |  |
| *Chondrostoma nasus* | 2.0 ±0.00 | prey |  |  |
| *Clupeonella cultriventris* | 3.0 ±0.0 | prey |  |  |
| *Cobitis elongata* | 3.3 ±0.4 | prey |  |  |
| *Cobitis elongatoides* | 3.3 ±0.4 | prey |  |  |
| *Cobitis strumicae* | 3.3 ±0.4 | prey |  |  |
| *Cobitis taenia* | 3.3 ±0.4 | prey |  |  |
| *Cobitis tanaitica* | 3.2 ±0.4 | prey |  |  |
| *Coregonus atterensis* | 3.4 ±0.3 | prey |  |  |
| *Coregonus bavaricus* | 3.3 ±0.5 | prey |  |  |
| *Coregonus danneri* | 3.3 ±0.5 | prey |  |  |
| *Coregonus hoferi* | 3.4 ±0.3 | prey |  |  |
| *Coregonus renke* | 3.4 ±0.3 | prey |  |  |
| *Cottus gobio* | 3.2 ±0.2 | prey |  |  |
| *Cottus haemusi* | n.i. | n.i. |  |  |
| *Cottus metae* | 3.3 ±0.5 | prey |  |  |
| *Cottus poecilopus* | 3.0 ±0.34 | prey |  |  |
| *Cottus rhenanus* | 3.2 ±0.5 | prey |  |  |
| *Cottus transsilvaniae* | 3.2 ±0.5 | prey |  |  |
| *Cyprinus carpio* | 3.1 ±0.0 | prey |  |  |
| *Dicentrarchus labrax* | 3.5 ±0.50 | prey |  |  |
| *Esox lucius* | 4.1 ±0.4 | predator |  |  |
| *Eudontomyzon danfordi* | 3.2 ±0.53 | prey |  |  |
| *Eudontomyzon mariae* | 3.4 ±0.5 | prey |  |  |
| *Eudontomyzon vladykovi* | 3.6 ±0.4 | prey |  |  |
| *Gasterosteus aculeatus* | 3.3 ±0.2 | prey |  |  |
| *Gasterosteus gymnurus* | 3.3 ±0.2 | prey |  |  |
| *Gobio carpathicus* | 3.2 ±0.4 | prey |  |  |
| *Gobio gobio* | 3.1 ±0.38 | prey |  |  |
| *Gobio obtusirostris* | n.i. | n.i. |  |  |
| *Gymnocephalus ambriaelacus* | 3.4 ±0.4 | prey |  |  |
| *Gymnocephalus baloni* | 3.5 ±0.37 | prey |  |  |
| *Gymnocephalus cernua* | 3.3 ±0.57 | prey |  |  |
| *Gymnocephalus schraetser* | 3.4 ±0.43 | prey |  |  |
| *Hucho hucho* | 4.2 ±0.74 | predator |  |  |
| *Huso huso* | 4.4 ±0.3 | predator |  |  |
| *Knipowitschia caucasica* | 3.3 ±0.45 | prey |  |  |
| *Lampetra fluviatilis* | 4.5 ±0.80 | predator |  |  |
| *Lampetra planeri* | 4.1 ±0.7 | predator |  |  |
| *Leucaspius delineatus* | 3.2 ±0.37 | prey |  |  |
| *Leuciscus idus* | 3.8 ±0.59 | prey |  |  |
| *Leuciscus leuciscus* | 2.9 ±0.1 | prey |  |  |
| *Liza aurata* | 2.8 ±0.33 | prey |  |  |
| *Liza ramada* | 2.3 ±0.20 | prey |  |  |
| *Liza saliens* | 2.9 ±0.38 | prey |  |  |
| *Lota lota* | 3.8 ±0.2 | prey |  |  |
| *Mesogobius batrachocephalus* | 4.2 ±0.73 | predator |  |  |
| *Misgurnus fossilis* | 3.4 ±0.45 | prey |  |  |
| *Mugil cephalus* | 2.5 ±0.17 | prey |  |  |
| *Neogobius fluviatilis* | 3.4 ±0.1 | prey |  |  |
| *Neogobius melanostomus* | 3.3 ±0.1 | prey |  |  |
| *Pachychilon pictum* | 2.8 ±0.30 | prey |  |  |
| *Pelecus cultratus* | 3.6 ±0.53 | prey |  |  |
| *Perca fluviatilis* | 4.4 ±0.0 | predator |  |  |
| *Petroleuciscus borysthenicus* | 3.1 ±0.35 | prey |  |  |
| *Petromyzon marinus* | 4.4 ±0.85 | predator |  |  |
| *Phoxinus phoxinus* | 3.2 ±0.4 | prey |  |  |
| *Platichthys flesus* | 3.3 ±0.2 | prey |  |  |
| *Ponticola eurycephalus* | 3.4 ±0.5 | prey |  |  |
| *Ponticola kessleri* | 3.5 ±0.53 | prey |  |  |
| *Ponticola syrman* | 3.4 ±0.48 | prey |  |  |
| *Proterorhinus semilunaris* | n.i. | n.i. |  |  |
| *Pungitius platygaster* | 3.5 ±0.37 | prey |  |  |
| *Pungitius pungitius* | 3.3 ±0.1 | prey |  |  |
| *Rhodeus amarus* | 3.0 ±0.34 | prey |  |  |
| *Romanichthys valsanicola* | 3.2 ±0.40 | prey |  |  |
| *Romanogobio antipai* | 3.3 ±0.4 | prey |  |  |
| *Romanogobio kesslerii* | 3.3 ±0.4 | prey |  |  |
| *Romanogobio uranoscopus* | 3.3 ±0.39 | prey |  |  |
| *Romanogobio vladykovi* | 3.4 ±0.4 | prey |  |  |
| *Rutilus frisii* | 3.6 ±0.0 | prey |  |  |
| *Rutilus heckelii* | n.i. | n.i. |  |  |
| *Rutilus meidingeri* | 3.4 ±0.50 | prey |  |  |
| *Rutilus rutilus* | 3.0 ±0.0 | prey |  |  |
| *Rutilus virgo* | n.i. | n.i. |  |  |
| *Sabanejewia balcanica* | 3.2 ±0.4 | prey |  |  |
| *Sabanejewia bulgarica* | 3.2 ±0.4 | prey |  |  |
| *Sabanejewia romanica* | 3.2 ±0.4 | prey |  |  |
| *Sabanejewia vallachica* | n.i. | n.i. |  |  |
| *Salmo dentex* | 3.5 ±0.5 | prey |  |  |
| *Salmo labrax* | 3.6 ±0.5 | prey |  |  |
| *Salmo schiefermuelleri* | 3.5 ±0.5 | prey |  |  |
| *Salmo trutta* | 3.4 ±0.1 | prey |  |  |
| *Salvelinus evasus* | 3.7 ±0.5 | prey |  |  |
| *Salvelinus umbla* | 3.9 ±0.5 | prey |  |  |
| *Sander lucioperca* | 4.0 ±0.78 | predator |  |  |
| *Sander volgensis* | 4.1 ±0.66 | predator |  |  |
| *Scardinius plotizza* | n.i. | n.i. |  |  |
| *Scardinius racovitzai* | n.i. | n.i. |  |  |
| *Silurus glanis* | 4.4 ±0.2 | predator |  |  |
| *Squalius cephalus* | 2.7 ±0.1 | prey |  |  |
| *Syngnathus abaster* | 3.2 ±0.40 | prey |  |  |
| *Telestes souffia* | 3.4 ±0.42 | prey |  |  |
| *Thymallus thymallus* | 3.1 ±0.42 | prey |  |  |
| *Tinca tinca* | 3.7 ±0.0 | prey |  |  |
| *Umbra krameri* | 3.3 ±0.39 | prey |  |  |
| *Vimba vimba* | 3.3 ±0.1 | prey |  |  |
| *Zingel streber* | 3.5 ±0.37 | prey |  |  |
| *Zingel zingel* | 3.8 ±0.46 | prey |  |  |
