# Supplementary material for: Incorporating ecological functions in conservation decision making
Source: Ecol Evol. 2017 Sep 7;7(20):8273–81. doi: 10.1002/ece3.3353 (PMC5648659; doi:10.1002/ece3.3353)
Supplement: Supplementary file 2 [file ECE3-7-8273-s002.docx]

**Appendix S2: Number of species and their occurrence rate in planning units that could not achieve the target.**

| **number of species** | **occurrence in pu** |
| --- | --- |
| 10 | 1 |
| 8 | 2 |
| 8 | 3 |
| 2 | 4 |
| 1 | 5 |
| 2 | 6 |
| 1 | 7 |
| 1 | 8 |
| 1 | 9 |
