# Supplementary material for: Incorporating ecological functions in conservation decision making
Source: Ecol Evol. 2017 Sep 7;7(20):8273–81. doi: 10.1002/ece3.3353 (PMC5648659; doi:10.1002/ece3.3353)
Supplement: Supplementary file 3 [file ECE3-7-8273-s003.docx]

**Appendix S3:** **Cohen's kappa value for all targets and weightings.** Trophic weighting is ratio highest to lowest with 3 being a weighting of 0.3-1, 4 being 0.25-1, 5 being 0.2-1, 6 being 0.15-1, 10 being 0.1-1, 20 being 0.05-1 and 100 being 0.01-1.

| **trophic weighting** | **target 3** | **target 5** | **target 7** | **target 10** |
| --- | --- | --- | --- | --- |
| **3** | 0.540 | 0.570 | 0.646 | 0.646 |
| **4** | 0.567 | 0.610 | 0.587 | 0.587 |
| **5** | 0.567 | 0.570 | 0.552 | 0.552 |
| **6** | 0.594 | 0.622 | 0.552 | 0.552 |
| **10** | 0.621 | 0.570 | 0.675 | 0.675 |
| **20** | 0.567 | 0.587 | 0.552 | 0.552 |
| **100** | 0.621 | 0.552 | 0.552 | 0.552 |
